# Changes in salivary oxytocin levels and bonding disorder in women from late pregnancy to early postpartum: A pilot study

**DOI:** 10.1371/journal.pone.0221821

**Published:** 2019-09-03

**Authors:** Eri Shishido, Takuya Shuo, Kaori Takahata, Shigeko Horiuchi

**Affiliations:** 1 Graduate School of Nursing Science, St. Luke’s International University, Tokyo, Japan; 2 Hokuriku University, Ishikawa, Japan; 3 St. Luke’s International University, Tokyo, Japan; Universita Cattolica del Sacro Cuore Sede di Roma, ITALY

## Abstract

**Objectives:**

This pilot study aimed to 1) follow the longitudinal changes in the salivary oxytocin level of pregnant women from late pregnancy to early postpartum, 2) examine the factors related to these changes, and 3) clarify the association of these changes with mother-infant bonding.

**Methods:**

This study used a longitudinal observational design and questionnaires to obtain objective and subjective data. For oxytocin evaluation, saliva samples were collected and their oxytocin levels were measured at 4-time points [i.e., **1**) 36–37 gestation weeks, **2**) 38–39 gestation weeks, **3**) 1–2 days postpartum, **4**) 4–5 days postpartum]. The oxytocin level was assayed in duplicates by enzyme-linked immunosorbent assay. Baseline data were evaluated using the Parental Bonding Instrument (25 items), State Trait Anxiety Inventory (20 items), and Center for Epidemiologic Studies Depression Scale. Postpartum data were evaluated using the Mother to Infant Bonding Scale Japanese Version (10 items), Maternity Blues Scale (13 items), and ‘Fatigue after Childbirth’ using the Visual Analogue Scale (VAS: 0–100 mm).

**Results:**

The participants were 13 primiparas with a mean age of 33 years. They had no depression or anxiety at the baseline. Their mean salivary oxytocin levels significantly increased from late pregnancy (36–39 gestation weeks) up to 1 day postpartum and then decreased until 5 days postpartum. There was a negligible correlation between the bonding disorder and the salivary oxytocin level on the 5th day after childbirth. A moderate correlation was observed between the maternity blues score and the salivary oxytocin level. There was a significant negative correlation between the postpartum fatigue and the salivary oxytocin level 1 day and 5 days after childbirth.

**Conclusion:**

The mean salivary oxytocin levels significantly increased from the baseline up to 1 day postpartum and then decreased until 5 days postpartum. The salivary oxytocin level was moderately associated with maternity blues and significantly with postpartum fatigue.

## Introduction

Elevated oxytocin levels during pregnancy have been shown to be associated with child rearing behavior, mother-infant bonding [1], and depletions with postpartum depression [2]. Feldman et al. (2007) reported that an increase in the plasma oxytocin level in early and late pregnancy was significantly and positively correlated with mother-infant bonding. However, epidural anesthesia and exogenous oxytocin used for inducing labor have a tendency to depress endogenous oxytocin and have been associated with mother-infant bonding interference and mothers’ depressed mood [3, 4, 5, 6]. In Japan, the rate of induced labor and labor promotion is 23.8%, and the proportion of Japanese women who receive synthetic oxytocin is 20% [7]. In Australia, the proportion of women who have induced or augmented labor is 45.3% (Department of Health, 2011) [8]. Therefore, special attention should be given to establishing support for the development and maintenance of healthy levels of endogenous oxytocin among women who are bearing children.

The changes in the plasma oxytocin level during pregnancy have been found to either vary from the early stage to the late stage or remain stable. It has been pointed out that individual differences are large and that no consensus has been obtained [9, 1]. Oxytocin levels can be measured in the saliva, plasma, cerebrospinal fluid, and urine depending on research aim and protocol. Among these, the validity of oxytocin measurement in the urine has not yet been clarified, and oxytocin has been mainly measured in the saliva and plasma [10].

As for behavioral epigenetics, oxytocin has been reported to exert its effect by binding to the oxytocin receptor. Epigenetic regulation of the oxytocin receptor gene (OXTR) has been demonstrated [11]. However, consistent results regarding the relation between OXTR and maternal behavior after childbirth have not yet been obtained [12, 13].

Quantification of the oxytocin level in the collected saliva is considered to be a measurement method with less physical invasion and burden on the subject [14]. Two previous studies have shown the correlation between salivary oxytocin level and plasma oxytocin level targeting perinatal women. Both studies involved postpartum women with the preintervention baseline oxytocin levels showing a moderate correlation (r = 0.41–0.59) [15, 16]. Thus, oxytocin plays a critical role in pregnancy and child rearing. However, there are apparently no studies of changes in the salivary oxytocin levels from pregnancy to postpartum. Initially, it is considered necessary to study the feasibility of establishing the relationship between changes in salivary oxytocin levels and mother’s mood and bonding behaviors before conducting a randomized controlled study. Overall, the essential aim is to maintain healthy levels of endogenous oxytocin during pregnancy and childbirth. Verification of these individual transitions provide important basic data.

This pilot study aimed to 1) follow the longitudinal changes in salivary oxytocin level of pregnant women from late pregnancy to early postpartum, 2) examine the factors related to these changes, and 3) clarify the association of these changes with mother-infant bonding.

## Materials and methods

### Study design

This study used a longitudinal observational design and questionnaires to obtain objective and subjective data. For oxytocin evaluation, saliva samples were collected and measured at 4-time points (i.e., measurement time points **1**): 36–37 gestation weeks, **2**) 38–39 gestation weeks, **3**) 1–2 days postpartum, and **4**) 4–5 days postpartum).

### Participants and setting

The participants included low-risk pregnant women of singleton cephalic presentation pregnancies 1) who were planning vaginal delivery (i.e., with or without epidural anesthesia), 2) who were between 36 and 37 gestation weeks before labor onset, 3) who were between 20 and 40 years old, and 4) who could communicate, read, and write in Japanese.

The exclusion criteria included the following: 1) obstetric history (e.g., cesarean section, preterm delivery), 2) undecided on the delivery method (i.e., with or without epidural anesthesia), 3) medical history (e.g., endocrine disease, mental disorder, epilepsy), 4) obstetric complications (e.g., pregnancy-induced hypertension, gestational diabetes mellitus, imminent abortion), 5) infection (e.g., HIV-, HBV-, HCV-positive), 6) alcohol addiction, 7) smokers, 8) breastfeeding, 9) illicit drug use, 10) oral cavity bleeding (e.g., periodontal disease, stomatitis), 11) brought children to maternity check-up.

There was no need to calculate the sample size as this pilot study aimed to provide a descriptive evaluation of the feasibility of the protocol. As this pilot study aimed to obtain objective and subjective data using a longitudinal study design and questionnaires as protocol, an estimated sample size at this stage was deemed sufficient. Thus, about 5 participants for each group were considered appropriate. Women who had an unplanned cesarean section were eligible and included in the study.

Pregnant women who met the eligible criteria were selected from the medical records. When eligible pregnant women at 34 gestation weeks visited for maternity check-up, we first verbally explained our research using an explanatory booklet containing information about the provision of confidentiality and anonymity of their data. Women also received a refusal form at that time indicating that they could withdraw from the study any time without any disadvantage. After obtaining written informed consent, the participants were consulted regarding their first experiment day (36–37 gestation weeks). At the end of the experiment, all the participants received QUO cards (about $20 per time) as remuneration.

This pilot study was conducted at a birth center in Tokyo, Japan between December 2017 and April 2018. The Institutional Review Board of St. Luke’s International University, Tokyo, Japan approved the study protocol (17–A063).

### Procedures

The number of uterine receptors reaches its peak at 39 gestation weeks, thus oxytocin level is measured twice during pregnancy [11]. For this reason, it is important to check the baseline oxytocin level and determine whether it is elevated or not. Therefore, it is necessary to measure the baseline oxytocin level once before the peak level is reached. In this study, the salivary oxytocin level was measured at 4-time points: **1**) 36–37 gestation weeks (baseline), **2**) 38–39 gestation weeks, **3**) 1–2 days postpartum, and **4**) 4–5 days postpartum. Saliva was collected within the period from 8:00 to 12:00. The morning time was used to control for diurnal variation.

Based on the guidelines for quantifying oxytocin from salivary samples (Salimetrics LCC, 2015), the participants were asked not to drink alcohol or caffeine and not to see a dentist before the experiment day. They were also instructed to finish their meals, brush their teeth 1 hour before the experiment, and not to use lipsticks and lip balm [17]. They were asked to keep their mobile phones during the experiment as calls could change their mood.

The experiment was carried out in a quiet private room in the birth center. The participants rinsed their mouth with water to prevent contamination of their saliva. They watched *an instructional video of saliva collection* (about 4 minutes), and drank 100 mL of water. After 20 minutes from wearing the headphones and watching a DVD movie (*Serenity of the River*), the first saliva sample was collected. The saliva collection method was repeated 3 times in the following sequence: 1 minute for saliva pooling, 1 minute for saliva collection, and 30 seconds for a break. To increase the amount of saliva collected, the participants let their saliva pool while viewing photos of lemon and plums. At each measurement point, at least 2.0 mL of saliva was collected in a polypropylene tube by passive drool after pooling the saliva in the mouth for 1 minute. When the volume was less than 2.0 mL, saliva was recollected as in the first attempt. The collected salivary samples were immediately stored in a freezer at –80°C (Cryo Porter CS-80C, Scinics Corp., Tokyo, Japan).

After saliva collection, the participants were asked to answer a questionnaire at 36–37 gestation weeks and twice after childbirth.

### Measurements

#### Measurement of salivary oxytocin level

For the primary objective, the salivary oxytocin level was measured at 4-time points as described earlier. The oxytocin level was assayed in duplicates by enzyme-linked immunosorbent assay (ELISA; ENZO Life Sciences, NY, USA) following the protocol of Carter et al. [18].

Aprotinin (500 KIU/μL) was added to the salivary samples after thawing them to prevent proteolytic degradation. As indicated in the ELISA manual, the intra-assay and inter-assay coefficients of variability are 12.6%-13.3% and 11.9%-20.9%, respectively (Product Manual Oxytocin ELISA kit). The oxytocin level was determined in duplicates by ELISA. These analyses were carried out by one of the authors (TS) in the School of Pharmacy at Hokuriku University in Japan.

### Questionnaires baseline data at 36–37 gestation weeks

Baseline data were evaluated using the Parental Bonding Instrument (PBI) (25 items), State Trait Anxiety Inventory (STAI) (20 items), and Center for Epidemiologic Studies Depression Scale (CESD) (20 items).

#### Parental Bonding Instrument

The PBI is a scale originally created by Parker et al. (1979) [19] for evaluating the child-rearing attitudes of parents who are carers. A Japanese version created by Ogawa (1991) [20] has confirmed the validity and reliability of PBI. The PBI is used for evaluating the caregiver’s child-rearing memories and nurturing experiences about caring for her child up to the age of 16 years. The PBI consists of 25 items as follows: ‘rearing care’, 12 items (care score) and ‘overprotection’, 13 items (overprotection score), and is evaluated by a four-point Likert scale from 0 (*completely different*) to 3 (*very much*). The higher the care score, the more the parents are evaluated as affectionate and accepting with a receptive attitude. The higher the overprotection score, the more the parents are evaluated as being engaged in overinterference and overprotection.

#### State-Trait Anxiety Inventory

Spielberger et al. (1970) [21] developed the STAI which is composed of two scales. One scale measures state anxiety, which indicates the strength of anxiety at the time the person is being tested. The other scale measures trait anxiety, which indicates a personality characteristic that is more of a long-term feature of the person. Nakazato et al. (1982) [22] developed the Japanese version of STAI.

In the present study, we measured trait anxiety to grasp the participant’s general personal anxiety characteristic. Trait anxiety is measured using a four-point Likert scale from 1 (*not at all*) to 4 (*absolutely*) and consists of 20 items. Higher scores indicate stronger anxiety. The STAI shows the criterion of discriminant and predictive validity, and the scale may be useful in research for measuring perinatal women’s anxiety [23].

#### Center for Epidemiologic Studies Depression Scale

Radloff (1977) [24] has developed the CESD and confirmed its reliability and validity. It has also been translated into a Japanese version (Shima et al. 1985). This scale is scored using a four-point Likert scale from 1 (*not at all*) to 4 (*absolutely*) and consists of 20 items. For score evaluation, < 16 points is evaluated as “normal”, 16–20 points as “lightly depressed”, 21–25 points as “moderately depressed”, and ≥ 26 points as “severely depressed”. Women with strong depression as indicated by CESD have been reported to have high plasma and salivary oxytocin levels (Holt-Lunstad et al., 2011), whereas women with depression during pregnancy have low plasma oxytocin levels [25].

### Data at 1–2 days and 4–5 days postpartum

Mother-infant bonding was evaluated using the Mother to Infant Bonding Scale Japanese Version *(*MIBSJ*)* (10 items), maternity blues using the Maternity Blues Scale (13 items), and ‘Fatigue after Childbirth’ using the Visual Analogue Scale (VAS:0–100 mm).

#### Mother to Infant Bonding Scale Japanese Version

The Mother to Infant Bonding Scale (MIBS) was developed by Taylor et al. (2005) [26], and the Japanese version (MIBSJ) by Yoshida et al. (2012) [27]. The MIBS has been validated as a screening assessment scale for mother-infant bonding disorders after childbirth [28]. This scale is scored using a four-point Likert scale from 1 to 4 and consists of 10 items. The cut-off is 4 points or more. It consists of two factors, namely, Lack of Affection and Anger and Rejection.

#### Stein’s Maternity Blues Scale

Stein (1980) [29] developed the Maternity Blues Scale. This scale consists of 13 items, and the score ranges from 0 to 26 points. A score of ≥ 8 points indicates maternity blues. In the present study, it was used to measure the mental state in early postpartum.

### Data analysis

The outcomes were descriptively analyzed. The changes in the salivary oxytocin levels were compared between the 4 measurement points. Additionally, the changes in the salivary oxytocin levels were statistically compared within the measurement points using the *t* test with a two-sided 5% level of significance. The correlation between the deltas (i.e., changes in the oxytocin level at every measurement point) and the mother-infant bonding score was analyzed.

Statistical analyses were performed using IBM SPSS Statistics (version 24.0; Static Base and Advanced Statistics, IBM Japan, Tokyo, Japan).

## Results

**Fig 1:**
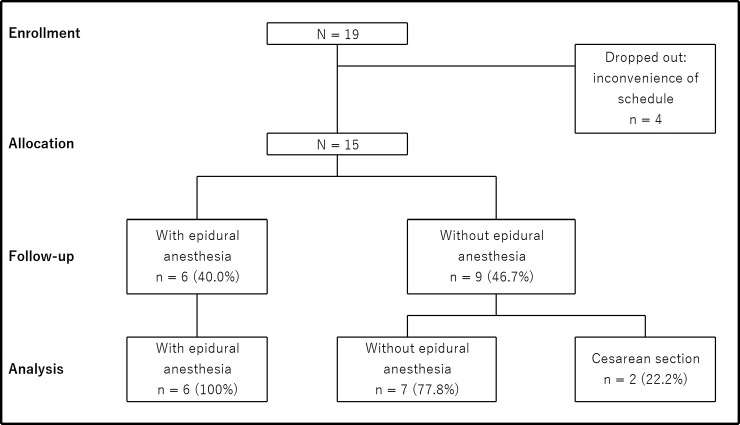
Participant flow diagram.

Written informed consent to participate in the study was obtained from 19 pregnant women. Of these, 4 women dropped out because of inconvenience of schedule, leaving 15 pregnant women for allocation and evaluation. The participant flow diagram is shown Fig 1.

### Characteristics of the participants

The demographic data and maternal outcomes of the participants are shown Table 1.

**Table 1:** Basic characteristics of the participants.

	With epidural anesthesia (n = 6)	Without epidural anesthesia (n = 7)	p-value
***Demographic data***			
Age	33.8 (SD 3.65)	33.1 (SD 3.02)	0.71
Gestation weeks	36.3 (SD 0.24)	36.5 (SD 0.39)	0.28
Primiparous	6 (100%)	7 (100%)	
Education > 12 years	6 (100%)	7 (100%)	
Married	6 (100%)	7 (100%)	
Living with partner	6 (100%)	7 (100%)	
CES-D > 16	0 (0.0%)	0 (0.0%)	
Anxiety: A-Trait	40.5 (SD 3.39)	44.1 (SD 4.33)	0.12
PBI (care)	28.6 (SD 6.56)	31.7 (SD 4.68)	0.35
PBI (overprotection)	8.66 (SD 9.13)	7.85 (SD 4.22)	0.83
***Maternal outcomes***			
Duration of labor (first stage)	477.5 (SD 127.5)	452.1 (SD 335.4)	0.66
Duration of labor (second stage)	115.1 (SD 78.3)	52.2 (25.5)	0.19
Administration of exogenous oxytocin (units)	17.5 (SD 9.8)	15.0 (SD 10.8)	0.35
Instrumental delivery	3 (50.0%)	2 (28.6%)	0.41
Bleeding (ml)	572.6 (SD 270.3)	463.1 (SD173.5)	0.39

CES-D, Center for Epidemiologic Studies Depression Scale

PBI, Parental Bonding Instrument

### Primary outcomes

#### Changes in salivary oxytocin levels

**Fig 2:**
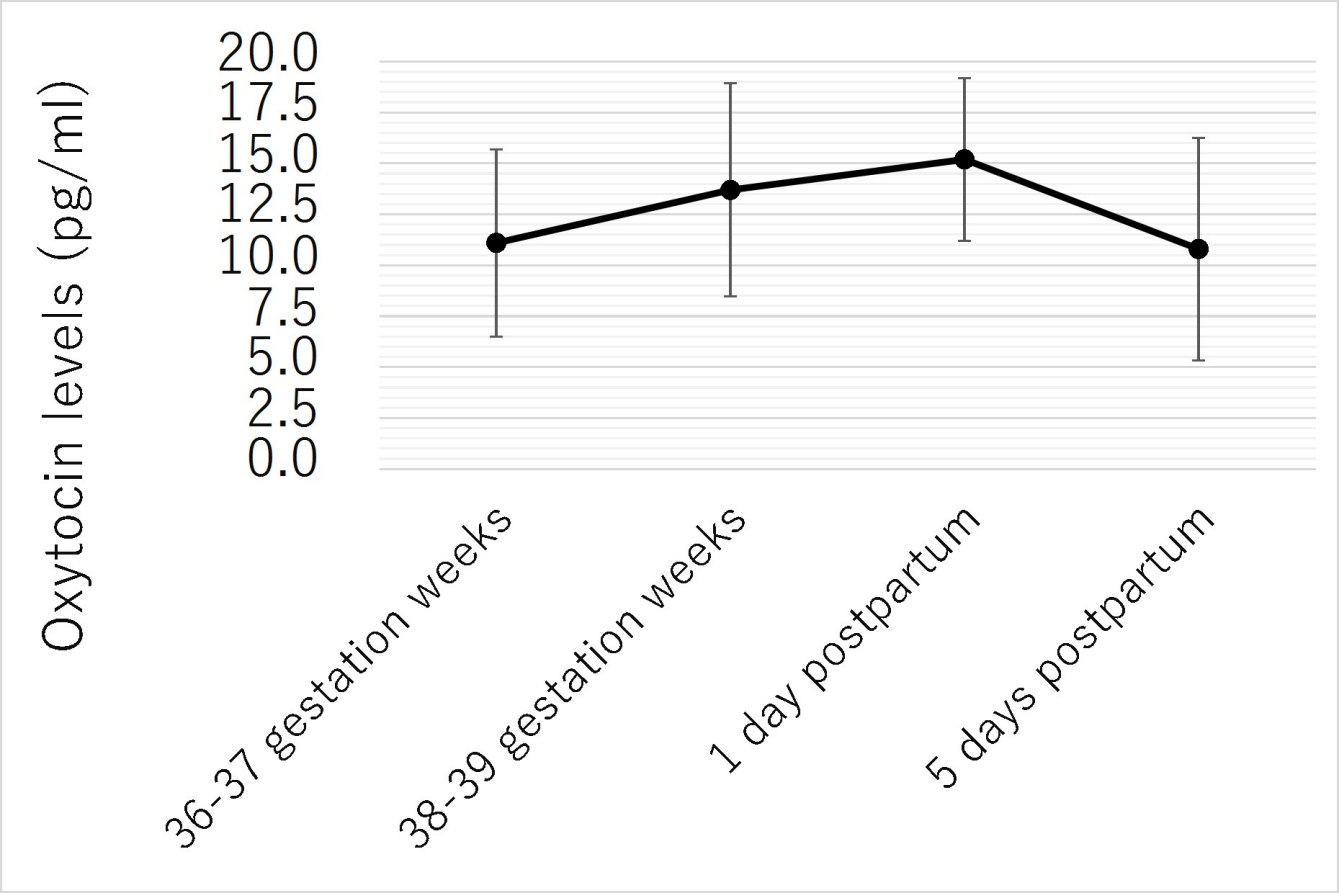
Changes in salivary oxytocin levels.

Of the 15 participants evaluated, 14 produced quantitatively analyzable salivary oxytocin levels. The changes in the mean salivary oxytocin levels (pg/ml) at the 4 measurement points were as follows: **1**) 11.1 (SD 4.60), **2**) 13.7 (SD 5.23), **3**) 15.2 (SD 4.00), and **4**) 10.8 (SD 5.46). The mean salivary oxytocin levels increased from late pregnancy (36–39 gestation weeks) up to 1 day postpartum, and then decreased until 5 days postpartum (Fig 2).

The salivary oxytocin level at the first measurement point was significantly lower than the salivary oxytocin level at the second and third measurement points (p = .01, p < .001). The salivary oxytocin level at the second measurement point was significantly higher than the salivary oxytocin level at the first and fourth measurement points (p = .01, p = .03). The salivary oxytocin level at the third measurement point was significantly higher than the salivary oxytocin level at the first and fourth measurement points (p < .001, p = .002). The salivary oxytocin level at the fourth measurement point was significantly lower than the salivary oxytocin level at the second and third measurement points (p = .03, p = .002).

#### Changes in salivary oxytocin levels: Without epidural anesthesia vs with epidural anesthesia

**Fig 3:**
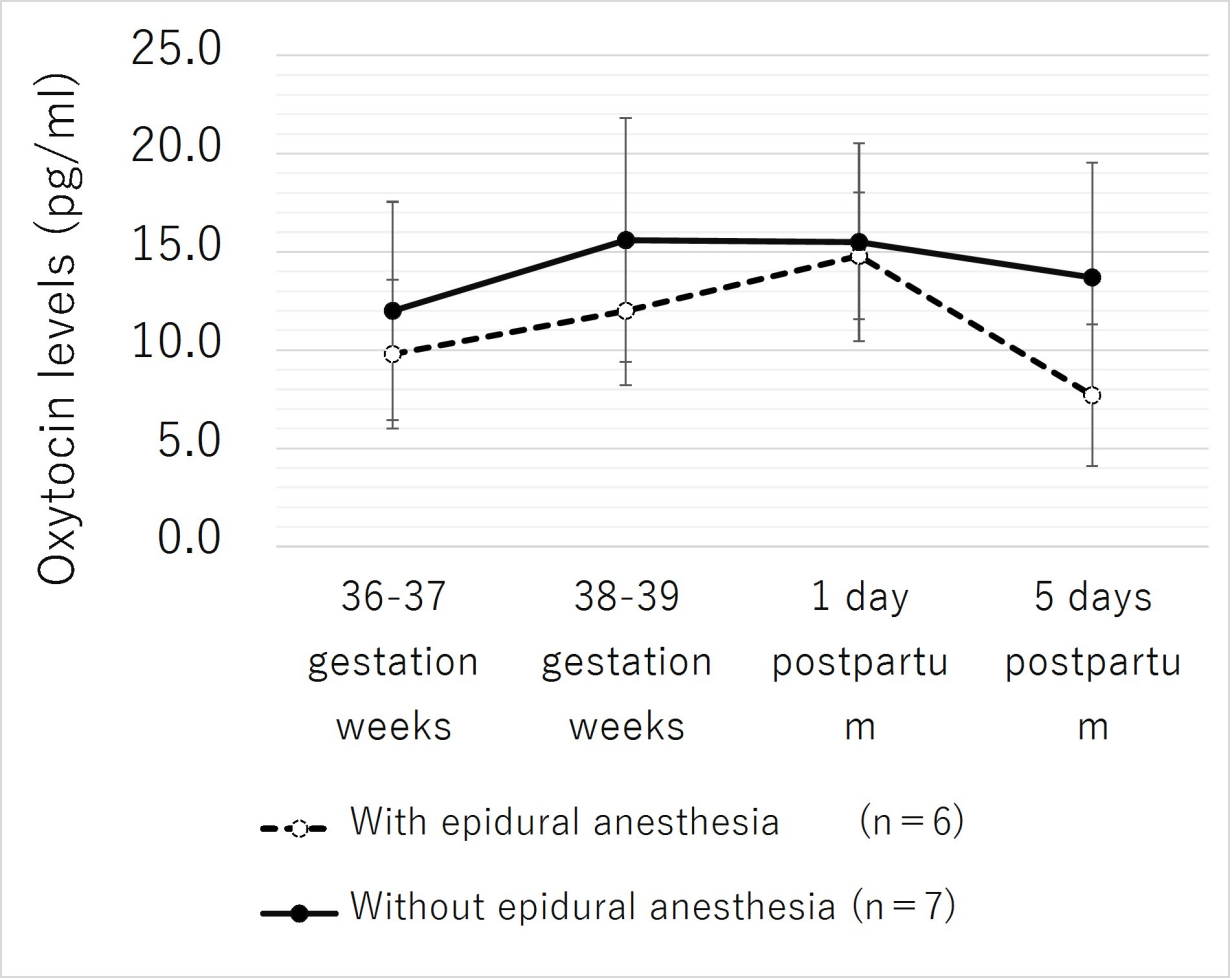
Changes in salivary oxytocin levels: Without epidural anesthesia vs with epidural anesthesia.

The mean salivary oxytocin level of the participants without epidural anesthesia was higher than the mean salivary oxytocin level of the participants with epidural anesthesia at all measurement points. There were no significant differences in the mean salivary oxytocin levels of the participants with epidural anesthesia at measurement points **1**), **2**), and **3**). The participants without epidural anesthesia had a significantly lower mean salivary oxytocin level than the participants with epidural anesthesia only at measurement point **4**) (p = .05) (Fig 3).

### Secondary outcomes

Table 2 shows a summary of the correlations between salivary oxytocin levels and bonding disorder, maternity blues, and postpartum fatigue. It indicates the r and p-values corresponding to the different postpartum days, salivary oxytocin levels, and bonding disorder.

**Table 2:** Correlations between salivary oxytocin levels and bonding disorder, maternity blues, and postpartum fatigue.

Correlation with mean salivary oxytocin level	Bonding disorder		Maternity blues		Postpartum fatigue	
	r	p-value	r	p-value	r	p-value
1–2 days postpartum	-0.25	0.38	0.07	0.81	-0.52	0.05
4–5 days postpartum	-0.21	0.47	-0.40	0.15	-0.68	0.007

### Salivary oxytocin level and bonding disorder

#### 1–2 days postpartum

The mean salivary oxytocin level of the participants (n = 3) during the puerperium period (1–2 days postpartum) judged to be associated with bonding disorder was 16.4 pg/ml (SD 3.33). The mean salivary oxytocin level of the participants (n = 10) judged to be normal (without bonding disorder) was 14.9 pg/ml (SD 4.25).

The salivary oxytocin level of the women judged to have bonding disorder was higher than the salivary oxytocin level of women judged to be normal, although the difference was not significant. The correlation between the bonding disorder score and the salivary oxytocin level 1–2 days postpartum was r = - 0.25, p = .38.

There was no correlation between each delta (the changes from 36–37 to 38–39 gestation weeks, and 38–39 gestation weeks to 1 day postpartum) and the mother-infant bonding score.

#### 4–5 days postpartum

The mean salivary oxytocin level of the participants (n = 6) during the puerperium period (4–5 days postpartum) judged to be associated with bonding disorder was 9.86 pg/ml (SD 5.27). The mean salivary oxytocin level of the participants (n = 7) judged to be normal was 11.6 pg/ml (SD 5.84).

The oxytocin level of the women judged to be associated with bonding disorder was lower than the oxytocin level of women judged to be normal, although the difference was not significant. The correlation between the bonding disorder score and the salivary oxytocin level 4–5 days postpartum was r = -0.21, p = .47.

There was no correlation between each delta (the changes from 36–37 to 38–39 gestation weeks, 38–39 gestation weeks to 1 day postpartum, and 1 day to 5 days postpartum) and the mother-infant bonding score.

### Salivary oxytocin level and maternity blues

#### 1–2 days postpartum

The mean salivary oxytocin level of the participants (n = 2) during the puerperium period (1–2 days postpartum) judged to be associated with maternity blues was 15.3 pg/ml (SD 3.81). The mean salivary oxytocin level of the participants (n = 11) judged to be normal was 15.2 pg/ml (SD 3.81), although no significant difference was observed.

The correlation between the maternity blues score and the salivary oxytocin level 1–2 days postpartum was r = 0.07, p = .81.

#### 4–5 days postpartum

The mean salivary oxytocin level of the participants (n = 5) during the puerperium period (4–5 days postpartum) judged to be associated with maternity blues was 9.16 pg/ml (SD 4.91). The mean salivary oxytocin level of the participants (n = 8) judged to be normal was 12.1 pg/ml (SD 5.83). There was no significant difference.

The correlation between the maternity blues score and the salivary oxytocin level 4–5 days postpartum was r = -0.40, p = .15. The higher the maternity blues score, the greater the tendency of the salivary oxytocin level to decrease.

### Salivary oxytocin level and postpartum fatigue

#### 1–2 days postpartum

The correlation between the postpartum fatigue score and the salivary oxytocin level 1–2 days postpartum was r = -0.52, p = .05. The higher the postpartum fatigue score, the more significant decrease in the oxytocin level.

#### 4–5 days postpartum

The correlation between the postpartum fatigue score and the salivary oxytocin level 4–5 days postpartum was r = -0.68, p = .007. Similarly to 1–2 days postpartum, the higher the postpartum fatigue score, the more significant decrease in the oxytocin level.

## Discussion

### Changes in salivary oxytocin levels

The mean salivary oxytocin levels were significantly higher at 38–39 gestation weeks than at 36–37 gestation weeks (p = .01). On the other hand, there was a significant decrease in the mean salivary oxytocin level on day 5 after childbirth compared with day 1 after childbirth (p = .002).

The number of oxytocin receptors in the uterus has been reported to peak at around 39 gestation weeks [11]. Studies by De Geest et al. (1985) showed that the plasma oxytocin level significantly increased with advancing gestational weeks [30]. Prevost et al. (2014) [9] reported a plasma oxytocin elevation rate of 73.2% from early pregnancy to late pregnancy. Stock et al. (1991) [31] reported a gradual increase in the plasma oxytocin level when approaching childbirth, and a decrease in the plasma oxytocin level until 8 weeks after childbirth. It has also been reported that the plasma oxytocin level during pregnancy hardly changes or that individual differences are large [1]. However, all these previous studies measured the plasma oxytocin levels. To the best of knowledge, there is as yet no study quantifying the salivary oxytocin level from late pregnancy to postpartum.

The correlation between the plasma, saliva, and systemic oxytocin levels in perinatal women was reported as r = 0.41–0.59 [15, 16], indicating a moderate correlation. In the present study, the oxytocin level was measured in the saliva. The salivary oxytocin level was elevated at 38–39 gestation weeks, consistent with the studies of De Geest et al. (1985), Prevost et al. (2014), and Stock et al. (1991).

### Without epidural anesthesia vs with epidural anesthesia

The participants with epidural anesthesia had a significantly lower salivary oxytocin level than the participants without epidural anesthesia on only 5 days postpartum (p = .05).

The factors associated with a decrease in the salivary oxytocin level in patients with epidural anesthesia 5 days postpartum were examined. Postpartum fatigue was suggested to have exerted an effect.

As a background, it was recognized that the salivary oxytocin level significantly decreased as the postpartum fatigue score increased both 1 day and 5 days postpartum. This correlation was particularly strong 5 days after childbirth.

In the study by Niwayama et al. (2017) of mood changes and salivary oxytocin levels during breastfeeding in 24 women [2], they showed an association between a decrease in the fatigue score and an increase in the salivary oxytocin level. These previous results are consistent with our present results.

### Salivary oxytocin level and bonding disorder

It is difficult to fairly judge any bonding disorder 1 day after childbirth because of the small amount of time spent with the baby in the same room at night and the short breastfeeding periods.

Bonding disorder is reported to occur in 15%-40% of postpartum cases. In mild bonding disorder, about half of the cases are affected on the first day and the other half are affected after 1 week of birth. Skin-to-skin-contact (STSC) has been identified as one of the prevention methods (Perinatal Mental Health Consensus Guide, 2017) [32, 33]. In several previous studies, STSC intervention resulted in a better maternal relationship, thereby decreasing the Edinburgh Postnatal Depression Scale (EPDS) score, cortisol concentration, and stress level [10, 34, 35].

In addition, compared with the control group (n = 52), the STSC group (n = 56) started breastfeeding immediately after childbirth. STSC intervention is considered to affect maternal and child-rearing aspects and breastfeeding [36].

In the present study, 2 participants (15.4%) were judged to have bonding disorder from the first day after childbirth. However, these 2 participants showed no decrease in the bonding disorder score even on the 5th day after childbirth and the bonding disorder remained. On the 5th day after childbirth, there were 6 participants who were judged as having bonding disorder (46.2%). There were 14 participants who performed STSC, which is considered to be one of the prevention methods for bonding disorder.

In this pilot study, the correlation between the bonding disorder score and the salivary oxytocin level was very weak. There is still no effective treatment for bonding disorder, and basic research on such disorder remains few. At this point, the use of salivary oxytocin level as an auxiliary tool for the definitive diagnosis of bonding disorder has yet to be fully established.

It is not yet clear how much the changes and deltas in the salivary oxytocin levels affect mother-infant bonding. However, a bonding disorder for 5 days postpartum may lead to a 1-month postpartum bonding disorder and abuse [37], thus this should be screened. Bonding disorder could be used as an indicator for screening if it is related to the changes and deltas in the oxytocin levels.

Additionally, since there are no consistent conclusions regarding the relation between mother-infant behavior and OXTR, further studies are needed to closely examine the relation between OXTR and mother-infant bonding.

### Salivary oxytocin level and maternity blues

The participants who were judged to have maternity blues had a lower mean salivary oxytocin level than the participants who were judged as normal. The correlation between the maternity blues score and the salivary oxytocin level 5 days after childbirth was r = - 0.40, p = .15. The higher the maternity blues score, the lower the mean salivary oxytocin level.

Maternity blues is a transient emotional instability that occurs 3–10 days postpartum, many of which do not require treatment. The frequency of the occurrence of maternity blues in Japan is reported to be about 10%-30%, and about 5% of those affected are reported to progress to postpartum depression [38].

In the present study, the incidence of maternity blues on the first day after childbirth was 15.4%, and that on the fifth day after childbirth was 38.5%. These results indicate that the incidence of maternity blues increased on the fifth day after childbirth.

Although changes in postpartum depression and various hormones (i.e., oxytocin, estrogen, cortisol, and vasopressin) have been investigated mainly using EPDS questionnaires, their specific relations remain to be established [39]. Studies of the relation between postpartum depression and plasma oxytocin levels have been conducted in 73 low-risk pregnant women. The plasma oxytocin level was measured twice in the second trimester of pregnancy (30–34 gestation weeks) and 2 weeks after childbirth [40]. The plasma oxytocin level in the second trimester of pregnancy was found to be a predictor of postpartum depression occurring within 2 weeks after childbirth. This suggests that the plasma oxytocin level may affect maternal behavior.

Regarding postpartum depression, it has been shown to be related to child abuse, child abandonment, or suicide when it becomes severe [41]. However, to the best of our knowledge, there are still no concrete studies of the relation between postpartum depression (or maternity blues) and salivary oxytocin level.

In Japan, women have to stay in the hospital for 4–6 days after childbirth. It is during this period that the peak onset of maternity blues occurs.

Considering that about 5% of the incidence of maternity blues progress to postpartum depression, a correct diagnosis of maternity blues is essential. In addition, there is also a need to clarify any association of maternity blues with the level of salivary oxytocin as a biological indicator and an auxiliary tool.

According to a study that showed the relation between postpartum depression and OXTR, it was suggested that epigenetic variation which decreases the expression of OXTR in a susceptible genotype may play a contributory role in the etiology of postpartum depression [42]. Therefore, in addition to oxytocin levels, the necessity of investigating more about OXTR has also been suggested.

### Salivary oxytocin level and postpartum fatigue

The factors related to the decrease in the salivary oxytocin levels of the participants with epidural anesthesia 5 days after childbirth were examined. Postpartum fatigue was suggested to be correlated with the salivary oxytocin level.

A good illustration of this correlation was the significant decrease in the salivary oxytocin level as the postpartum fatigue score became higher from 1 day to 5 days after childbirth. In particular, the correlation between postpartum fatigue and salivary oxytocin level was assessed as r = -0.68, p = .007. A strong correlation was observed in the participants with epidural anesthesia.

The results showed that the higher the postpartum fatigue score, the lower the salivary oxytocin level. Moreover, the less feeling of fatigue, the higher the salivary oxytocin level.

## Conclusions

The mean salivary oxytocin level significantly increased from the late gestation weeks (baseline) up to 1 day postpartum, and then decreased until 5 days postpartum. The salivary oxytocin level was found to be moderately associated with maternity blues and significantly associated with postpartum fatigue. These preliminary findings are recommended to be subsequently confirmed in future multicenter randomized studies involving a larger sample size.

## Supporting information

S1 File Baseline Japanease. (DOCX)

S2 File renamed_8344d. (DOCX)

S3 File Postpartum Japanease. (DOCX)

S4 File Postpartum English. (DOCX)
