# Supplementary material for: Changes in salivary oxytocin levels and bonding disorder in women from late pregnancy to early postpartum: A pilot study
Source: PLoS One. 2019 Sep 3;14(9):e0221821. doi: 10.1371/journal.pone.0221821 (PMC6719851; doi:10.1371/journal.pone.0221821)
Supplement: S1 File — (DOCX) [file pone.0221821.s001.docx]

|  |  |  |
| --- | --- | --- |

**Appendices 1.**

Baseline questionnaire (Japanese version)

)


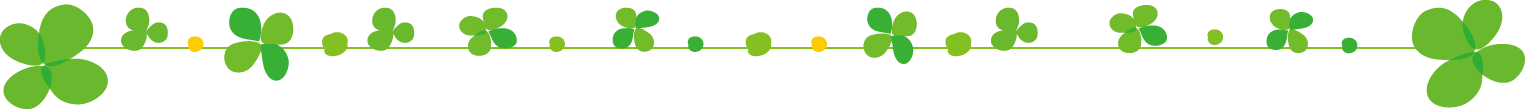


「妊婦の妊娠後期から産褥早期の唾液オキシトシン値の変化

とボンディング障害：予備研究」研究に、ご参加いただき、

ありがとうございます。

アンケート用紙は両面、2ページあり、10分ほどかかります。

このデータは、今回測定するホルモンとの関連を観察するために

使用させていただきます

　　　なお、質問の中には、一部ネガティブな表現が含まれる項目があります。

　　　もし回答にて心が不安定になる場合には、回答を中止して頂いてかまいません。


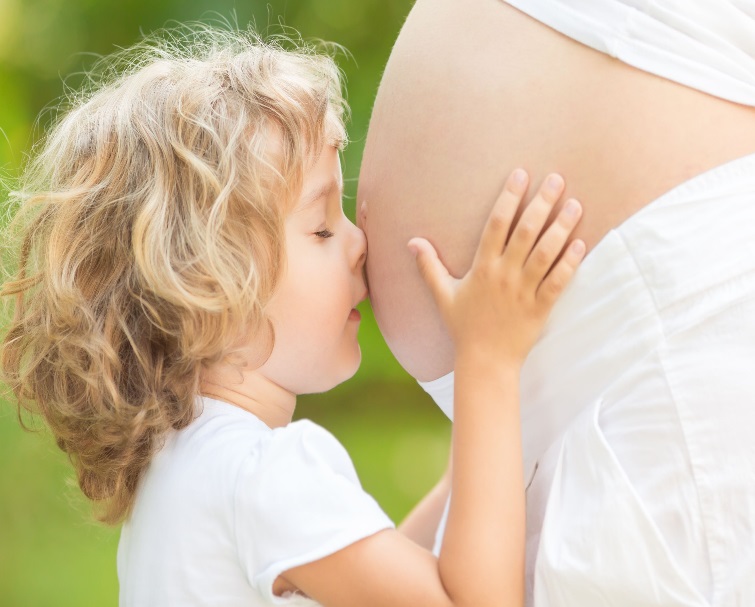


1. 心の状態についてお聞きします。

１）**この1週間の**、あなたの体や心の状態についてお聞きいたします。下の20項目の　　　文章を読み、あてはまる番号に、○を1つかこんでください。

裏に続きます♪♪

2）**普段、一般に**どの程度の状態か、当てはまる番号を、○で囲んでください。

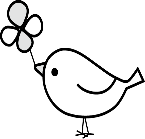


　　　　　　　　　　　　　　　　　　　　　　　　　　　　　　　　　　　あと半分です

1. あなたの両親の接し方や行動についてお聞きいたします。

あなたが**16歳まで**の、あなたの（父親・母親）について、覚えている範囲でもっとも適切だと思える番号に○をつけてください。

3．あなた自身について、下記の表に回答して下さい。

選択肢のあるものには、当てはまる番号を○で囲んで下さい。

お疲れ様でした。アンケートはこれで終了です。


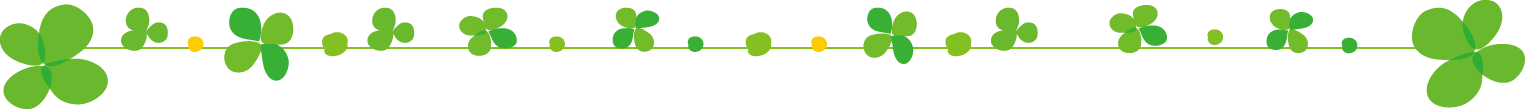
お手数ですが、回答の漏れがないが、再度ご確認をお願いいたします。
