# Supplementary material for: Changes in salivary oxytocin levels and bonding disorder in women from late pregnancy to early postpartum: A pilot study
Source: PLoS One. 2019 Sep 3;14(9):e0221821. doi: 10.1371/journal.pone.0221821 (PMC6719851; doi:10.1371/journal.pone.0221821)

|  |  |  |
| --- | --- | --- |

**Appendices 1.**

Baseline questionnaire (English version)


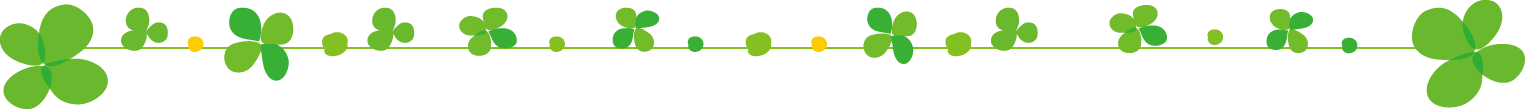


**“Changes in salivary oxytocin levels and bonding disorder in**

**women from late pregnancy to early postpartum: a pilot study”**

**Your cooperation would be highly appreciated.**

It takes about 10 minutes to answer this questionnaire.

This data is used to verify the association with the hormones.


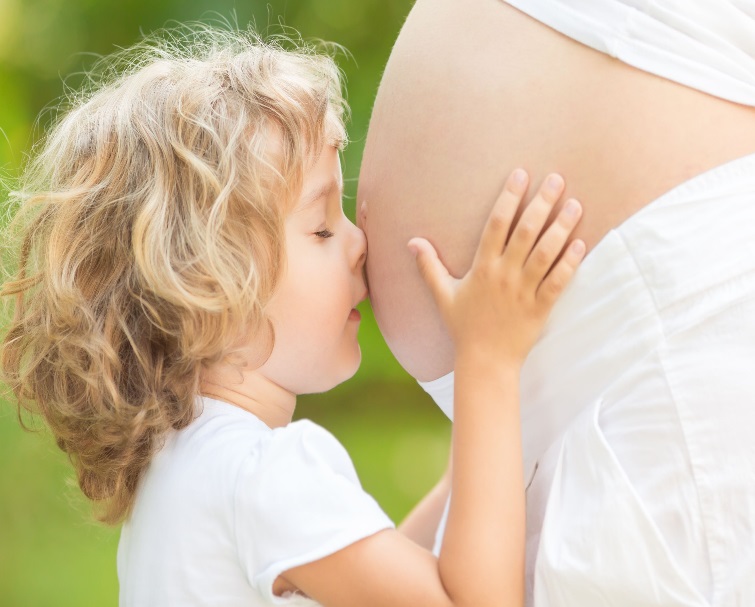


1. Below is a list of the ways you might have felt or behaved.

Please tell me how often you have felt this way during the past week.

2. How you generally feel? **Please select an applicable answer.**

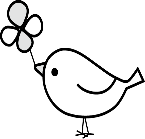


３．This questionnaire lists various attitudes and behaviors of parents. As you remember your mother and father in your first 16 years would you place a tick in the most appropriate box next to each question.

４．**Please select an applicable answer.**

Thank you for answering this questionnaire!!


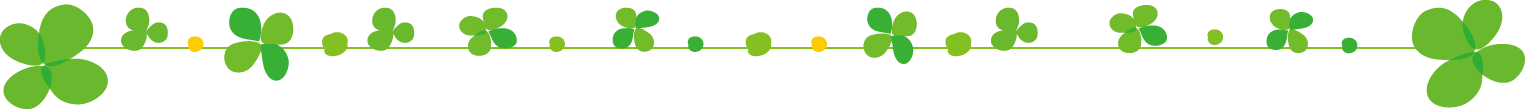

Supplement: S2 File — (DOCX) [file pone.0221821.s002.docx]
