# Supplementary material for: Changes in salivary oxytocin levels and bonding disorder in women from late pregnancy to early postpartum: A pilot study
Source: PLoS One. 2019 Sep 3;14(9):e0221821. doi: 10.1371/journal.pone.0221821 (PMC6719851; doi:10.1371/journal.pone.0221821)
Supplement: S3 File — (DOCX) [file pone.0221821.s003.docx]

|  |  |  |
| --- | --- | --- |

**Appendices 2.**

Postpartum questionnaire (Japanese version)

産後1日・産後5日　質問紙


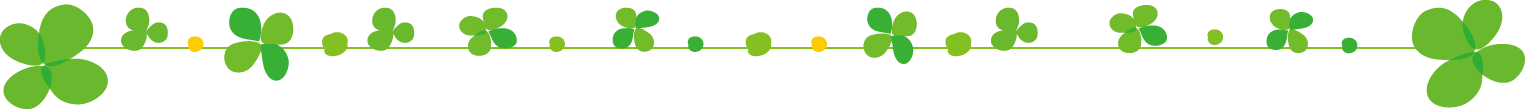


「妊婦の妊娠後期から産褥早期の唾液オキシトシン値の変化

とボンディング障害：予備研究」にご参加いただきありがとうございます。

ご出産おめでとうございます。

アンケート用紙は両面、1ページ半あり、10分ほどかかります。

このデータは、今回測定するホルモンとの関連を観察するために

使用いたします。


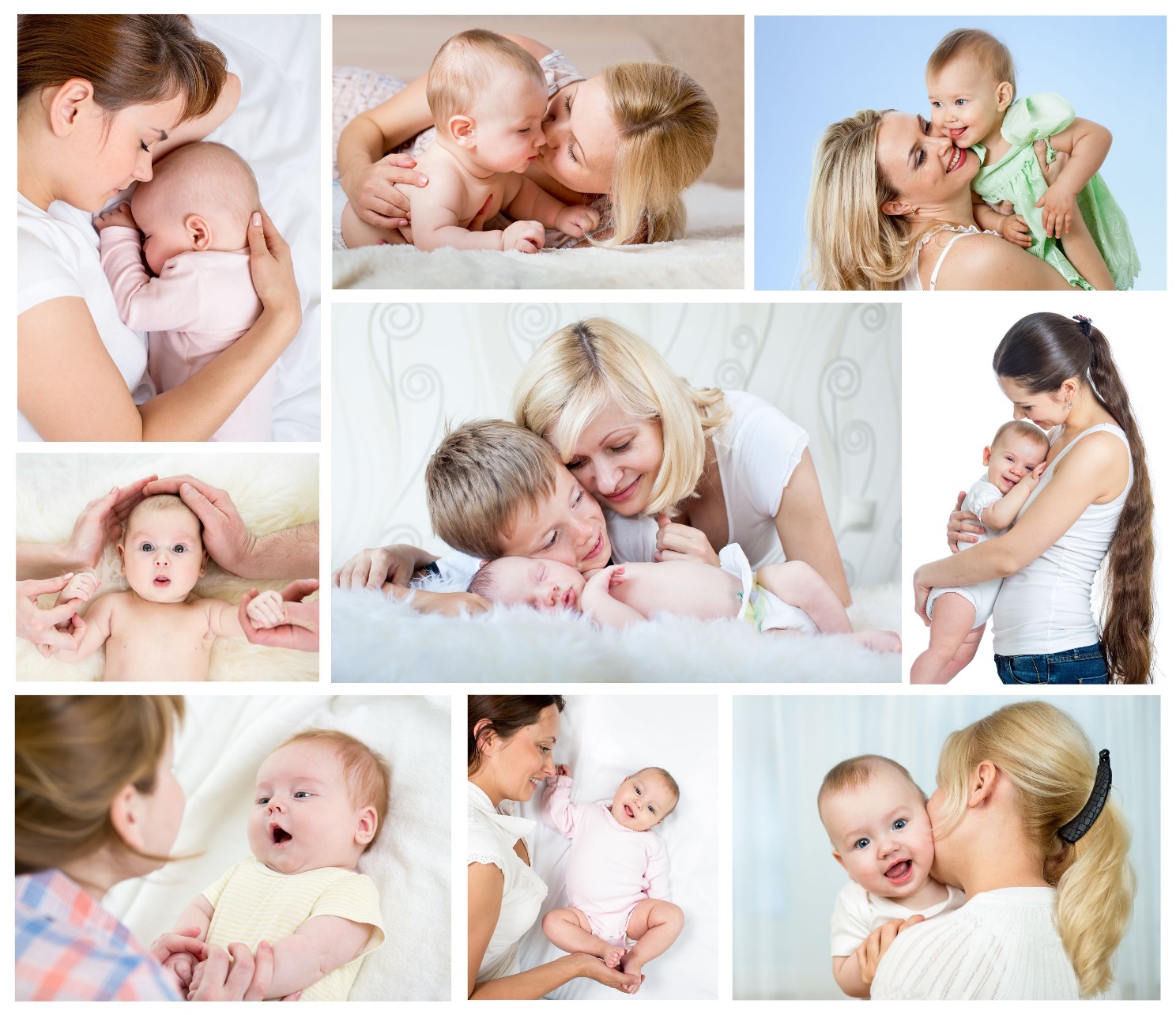


1．赤ちゃんついてお聞きします。

現在のあなたの赤ちゃんへの思いにあてはまるものに、○を1つ囲んでください。

2．**現在の**あなたの疲労感はどの程度ですか？

もっとも当てはまる場所に、**1か所**、**縦に線を**引いてください。

**＜例＞**0～100の中で１か所線を入れる

|  |  |  |  |  |  |  |  |  |  |
| --- | --- | --- | --- | --- | --- | --- | --- | --- | --- |
|  |  |  |  |  |  |  |  |  |  |

　　　　　０　　　　　　　　　　　　　　　　　　　　　　　１００

疲れていない←　　　　　　　　　　　　　　　　　　　　　→とても疲れている

３．**現在の**あなたの状態（A～H）にあてはまるものに、○を1つ囲んでください。

　　

４．**現在の**あなたの状態（I～M）について、「はい」または「いいえ」のいずれかに

○を囲んでください。

　　

お疲れ様でした。アンケートはこれで終了です。

お手数ですが、回答の漏れがないが、再度ご確認をお願いいたします。


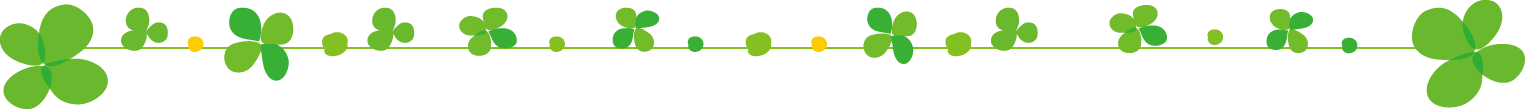
あと１回で研究は最後になりますので、ご協力よろしくお願いいたします。
