# Supplementary material for: Changes in salivary oxytocin levels and bonding disorder in women from late pregnancy to early postpartum: A pilot study
Source: PLoS One. 2019 Sep 3;14(9):e0221821. doi: 10.1371/journal.pone.0221821 (PMC6719851; doi:10.1371/journal.pone.0221821)

|  |  |  |
| --- | --- | --- |

**Appendices 2.**

Postpartum questionnaire (English version)


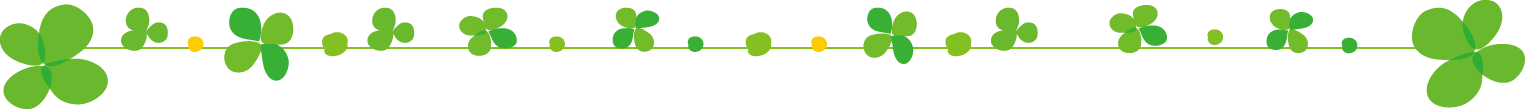


**“Changes in salivary oxytocin levels and bonding disorder in**

**women from late pregnancy to early postpartum: a pilot study”**

Thank you for participating in my study.

It takes about 10 minutes to answer this questionnaire.

This data is used to verify the association with the hormones.


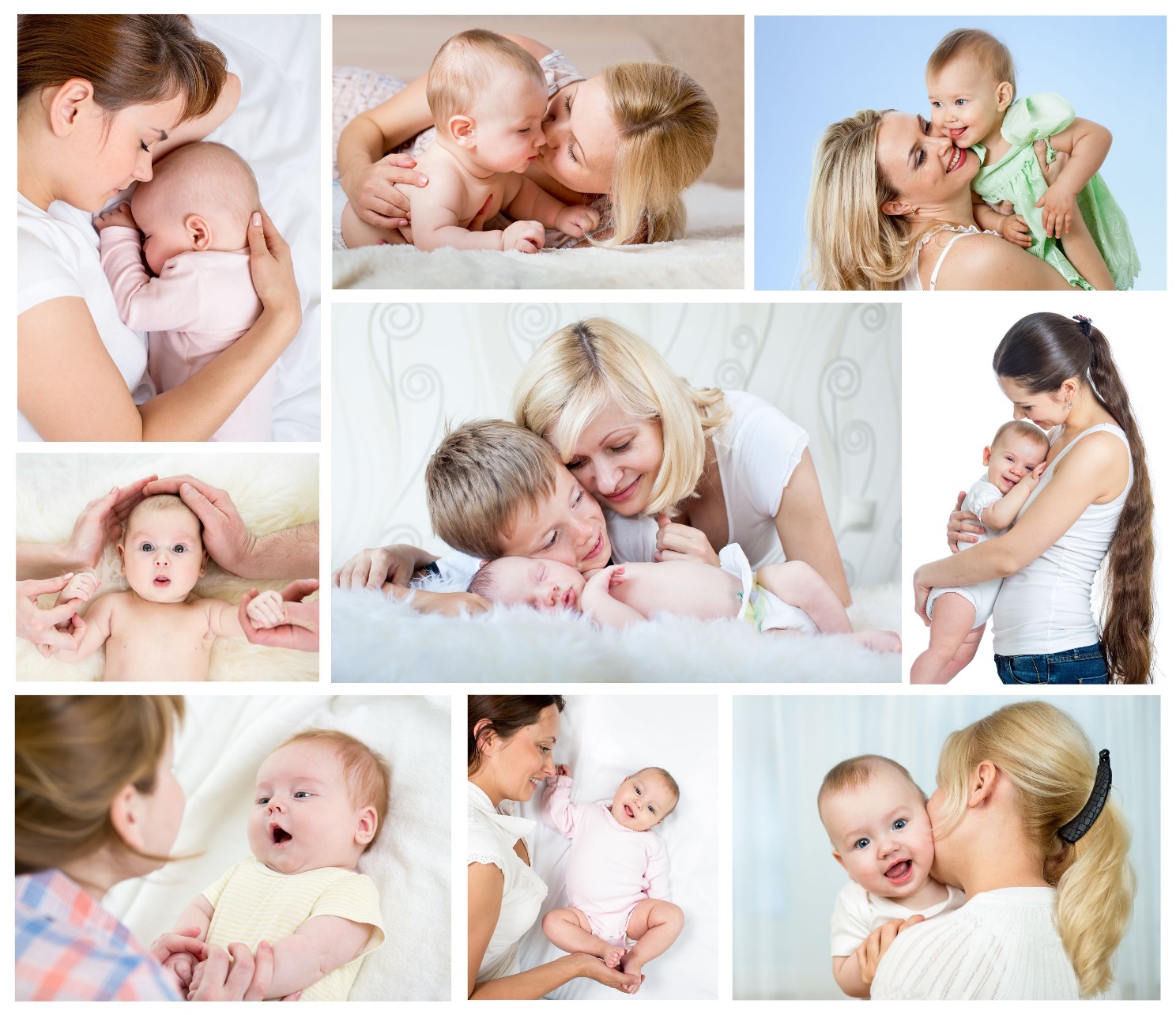


1．Please tell us how you feel about your baby. **Please select an applicable answer.**

2．How tired are you now? **Please write an applicable answer.**

**＜Example＞** Please draw a line vertically.

|  |  |  |  |  |  |  |  |  |  |
| --- | --- | --- | --- | --- | --- | --- | --- | --- | --- |
|  |  |  |  |  |  |  |  |  |  |

　　　　　０　　　　　　　　　　　　　　　　　　　　　　　１００

**Not strongly fatigue**←　　　　　　　　　　　　　　　　　→**Strongly fatigue**

３．How are you feeling now ? Please choose the one（A～H）.

　
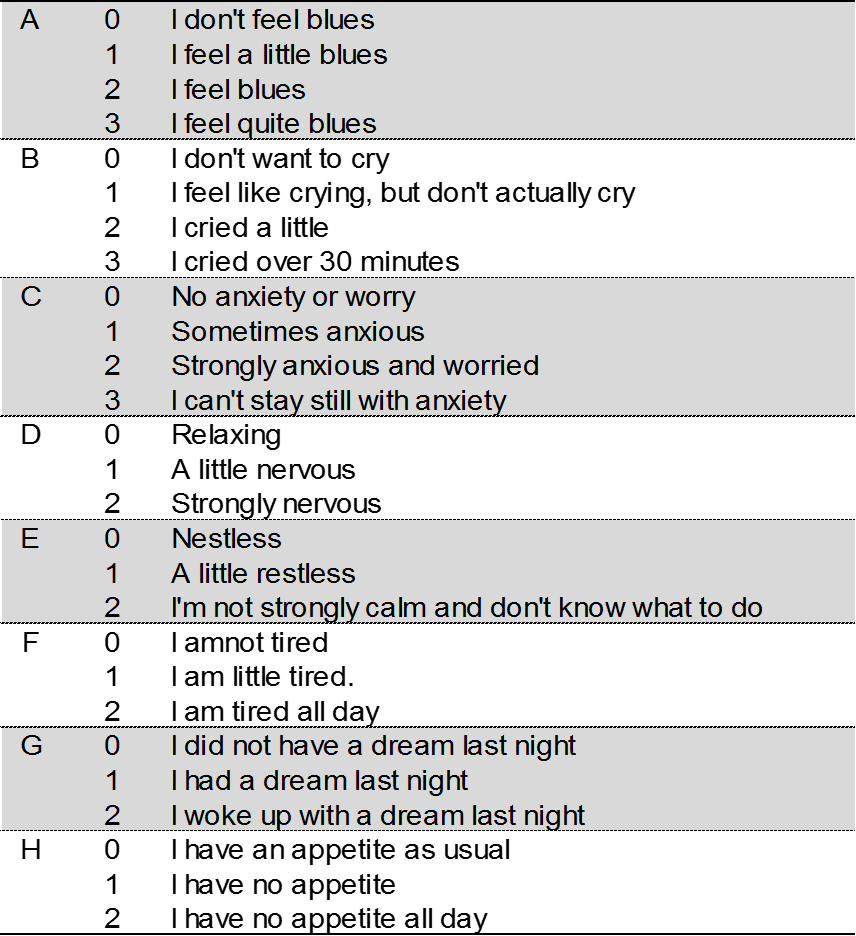


４．How are your health condition now ? Please choose one of these two（I～M）.

　

Thank you for answering this questionnaire!!


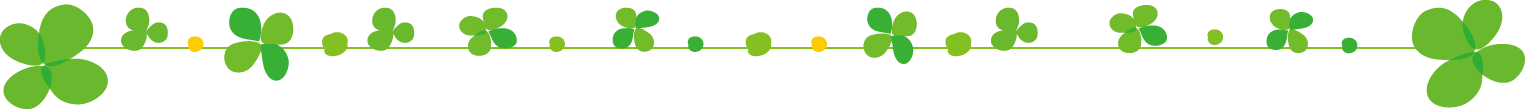

Supplement: S4 File — (DOCX) [file pone.0221821.s004.docx]
